# Outcomes after high-dose radiation in the management of neuroendocrine neoplasms

**DOI:** 10.1371/journal.pone.0252574

**Published:** 2021-06-02

**Authors:** Katherine S. Chen, Courtney Lawhn-Heath, Spencer Behr, Roxanna Juarez, Julia Whitman, Alan Paciorek, Eric K. Nakakura, Nicholas Fidelman, Mary Uan-Sian Feng, Emily K. Bergsland, Mekhail Anwar

**Affiliations:** 1 Department of Radiation Oncology, University of California San Francisco, San Francisco, CA, United States of America; 2 Department of Radiology, University of California San Francisco, San Francisco, CA, United States of America; 3 Helen Diller Family Comprehensive Cancer Center, University of California San Francisco, San Francisco, CA, United States of America; 4 Department of Epidemiology and Biostatistics, University of California San Francisco, San Francisco, CA, United States of America; 5 Department of Surgery, Division of Surgical Oncology, University of California San Francisco, San Francisco, CA, United States of America; 6 Department of Medicine, Division of Hematology/Oncology, University of California San Francisco, San Francisco, CA, United States of America; MD Anderson Cancer Center, UNITED STATES

## Abstract

**Background:**

Neuroendocrine neoplasms (NENs) comprise a rare and heterogenous group of cancers, for which the role of radiation therapy continues to evolve. The purpose of this study is to analyze oncologic outcomes after the use of high-dose radiation in management of NENs at a tertiary hospital.

**Materials and methods:**

We performed a retrospective review of patients who received high-dose radiation with intent to cure or provide durable local control (defined as biologically effective dose (BED) ≥40, α/β = 10) for a localized or metastatic NEN from 2006 to 2019. Evaluation of disease status after radiation was performed according to Response Evaluation Criteria in Solid Tumors (RECIST) criteria when possible. Patients were grouped by differentiation (well-differentiated (WD) or poorly-differentiated (PD)) and stage (localized/locally advanced disease (L) or metastatic (M)) in analysis of probabilities of progression after radiation.

**Results:**

45 patients completed a radiation course with BED ≥40 for a NEN (median BED 72). With a median follow-up of 24 months after radiation, the 2-year actuarial rates of local relapse-free survival, new metastasis-free survival, progression-free survival, and overall survival after radiation were 98%, 45%, 41%, and 69%, respectively. 25 patients (56%) developed new metastases after completion of radiation, including 33% (n = 3) of patients with WD-L disease, 44% (n = 8) of WD-M, 77% (n = 10) of PD-L, and 80% (n = 4) of PD-M, with progressively shorter median times to progression (26, 9, 8, and 3 months, respectively; p = 0.093). Of the 25 patients evaluable by RECIST, 68% (n = 17) achieved either a complete or partial best response in the irradiated lesion.

**Conclusions:**

These data suggest that focal, high-dose radiation has a role in the management of selected patients with NENs. Local failure is rare in patients with both well-differentiated and poorly-differentiated disease, although the predominant pattern of failure remains development of new metastases.

## Introduction

Neuroendocrine neoplasms (NENs) comprise a rare and heterogenous group of cancers, with widely varying aggressiveness and natural history based on primary site and tumor grade. As a result, treatment is often similarly heterogeneous and challenging. Consensus guidelines on management of NENs recommend a multidisciplinary approach to care, but there are many areas of treatment that remain controversial, such as the role of local therapy for high-grade or metastatic disease [1, 2].

While surgery is the mainstay of definitive treatment for localized neuroendocrine tumors, over half of patients may have unresectable disease at baseline or are not surgical candidates due to medical co-morbidities [3, 4]. Furthermore, 25% of patients may already have metastatic disease at the time of diagnosis [5, 6]. For patients with unresectable or metastatic disease, somatostatin analogues and targeted therapies are frequently employed as therapy to both provide symptomatic relief and stabilize disease. However, the duration of response is limited [7–9]. Given these ongoing challenges in oncologic control, there is a need for treatment that can provide adequate control of disease not amenable or ideal for surgery, as well as disease that progresses on systemic therapy.

Potentially, local control or ablation of tumor sites can (1) limit future morbidity from a growing lesion, (2) provide time off of systemic therapy (e.g. a “chemotherapy holiday”), (3) save next line systemic therapies for later progression, (4) address disease heterogeneity by targeting a subset of lesions not responding to current therapies, and (5) theoretically slow the seeding of metastatic disease by ablating a tumor deposit. Unfortunately, as many patients are not eligible for surgical management of their disease, a method for effective, personalized local treatment for NENs is needed.

Thus, in order to help fill these gaps in the treatment paradigm of NENs, the role of radiation therapy continues to evolve. External beam radiation therapy provides a non-invasive method to safely deliver local therapy to nearly any location in the body. In comparison to surgery, focal radiation therapy can confer low morbidity without significant recovery time, but local control and patient outcomes using newer techniques of radiation therapy are unknown. Moreover, with the success of peptide receptor radionuclide therapy (PRRT), radiation has been shown to be an effective modality for treatment of NENs [10]. Current evidence for the use of external radiation in NENs is scarce and largely comes from single-institution retrospective reviews with small sample sizes [11–16]. While radiation has long been used for palliation in the metastatic setting, the development of more modern, highly conformal radiotherapy techniques allows for the delivery of greater, more definitive doses of radiation not only to localized disease, but also to limited sites of metastatic disease without major toxicity. These increased doses may provide more durable local control and palliation as ablative doses are approached.

The purpose of this study is to analyze oncologic outcomes after the use of high-dose external beam radiation in management of NENs at a tertiary hospital.

## Materials and methods

At our institution, patients treated for NENs are included in a prospective clinical database. From this database, we identified 328 patients as having been referred to radiation oncology from 2006 to 2019. Patients treated for small cell lung cancer and Merkel cell carcinoma were excluded given their unique treatment paradigms. Using an α/β of 10 to represent tumor, the biologically effective dose (BED) for each course of radiation was calculated by the linear quadratic equation to help standardize between courses of varying fractionations. BED ≥40 was chosen to represent high-dose radiation treatment courses with intent to cure or provide durable local control. This threshold was designed to exclude traditional palliative radiation regimens, such as 30 Gy in 10 fractions. Of the original 328 patients seen in consultation, 61 patients went on to receive a course of high-dose radiation at our institution, and 45 of those had at least one follow-up scan and were included in this study. Approval for this study was provided by the University of California San Francisco Institutional Review Board, #14–14541. The need for patient consent was waived as this study was deemed minimal risk.

Evaluation of disease status after radiation was performed by two board-certified nuclear radiologists and one board-eligible nuclear radiologist according to Response Evaluation Criteria in Solid Tumors (RECIST) criteria to determine local recurrence at the irradiated site, presence of new metastases, and worsening of metastases known at the time of radiation consultation [17]. Baseline measurements were taken from the most immediate pre-radiation scan. The best response in the irradiated lesion after radiation was determined from subsequent imaging and classified as either complete response, partial response, stable disease, or progressive disease. Using the best response measurements as a new baseline, local recurrence was defined as an increase in lesion size that met RECIST criteria for progressive disease. For treated lesions not evaluable by RECIST, such as surgical bed sites without gross residual disease and bone lesions without soft tissue components, local recurrence was defined by radiologic review without RECIST.

IBM SPSS Statistics for Mac, version 26 (IBM Corp., Armonk, N.Y., USA) was used for the analysis. Due to the limited sample size, descriptive characteristics were primarily used to characterize patient and treatment characteristics and describe patterns of failure after high-dose radiation. For the purposes of analysis, patients were grouped by differentiation and stage. Disease was categorized as well-differentiated (WD) or poorly-differentiated (PD). Patients with localized or locally advanced disease (L) were grouped together, as separated from those with metastatic disease (M). In total, there were 4 groups: WD-L, WD-M, PD-L, PD-M. Using these groups, Kaplan-Meier log-rank tests were used to compare probabilities of progression after radiation. All endpoints were calculated from the end of radiation, and patients were censored at date of death or last follow-up. Acute and late toxicities following radiation were graded retrospectively according to Common Terminology Criteria for Adverse Events (CTCAE) version 5 [18]. Univariate binary logistic regression analysis was performed to identify factors associated with lesion response to radiation, local recurrence, and development of new metastases. For all analyses, an alpha level of <0.05 was considered statistically significant.

## Results

### Patient characteristics

45 patients with follow-up imaging completed a high-dose radiation course with BED ≥40 for their NEN at a median age of 57 years (interquartile range [IQR] 43–70). Baseline patient and treatment characteristics are summarized in Table 1. Most tumors were gastroenteropancreatic in origin (n = 13, 29%), with other primary sites including head and neck (n = 9, 20%), lung (n = 7, 16%), cervix (n = 4, 19%), other (n = 7, 16%), and unknown (n = 5, 11%). 60% of patients had well-differentiated neuroendocrine tumors (n = 27), and 40% of patients had poorly-differentiated neuroendocrine carcinomas (n = 18). At the time of radiation, 49% of patients had either localized or locally advanced disease (n = 22), while 51% had metastatic disease (n = 23).

**Table 1:** Baseline and treatment characteristics of patients who received high-dose radiation (n = 45).

Variable	Number of Patients (%)
Age	
<60	24 (53%)
≥60	21 (47%)
Gender	
Male	27 (60%)
Female	18 (40%)
Median KPS	90
Primary site	
Pancreas	9 (20%)
Head and neck	9 (20%)
Lung	7 (16%)
GI	4 (9%)
Cervix	4 (9%)
Other	7 (16%)
Unknown	5 (11%)
Grade	
1	9 (20%)
2	14 (31%)
3	19 (42%)
Unknown	3 (7%)
Differentiation	
Well-differentiated	27 (60%)
Poorly-differentiated	18 (40%)
Stage at RT	
Localized	6 (13%)
Locally advanced	16 (36%)
Metastatic	23 (51%)
Intent of RT	
Definitive	13 (29%)
Post-operative	8 (18%)
Oligoprogression	14 (31%)
Palliative	10 (22%)
Median BED (interquartile range)	72 (60–85)
Site treated	
Primary site	23 (51%)
Bone metastasis	9 (20%)
Nodal metastasis	6 (13%)
Other metastasis	7 (16%)

### Treatment characteristics

The median BED of the high-dose radiation courses was 72 (IQR 60–85), with example prescriptions ranging from 60 Gy in 3 fractions (BED 180) to 70 Gy in 33 fractions (BED 85) and 35 Gy in 14 fractions (BED 44). The intent of radiation was definitive (n = 13, 29%), post-operative (n = 8, 18%), for oligoprogression (n = 14, 31%), or purely for palliation (n = 10, 22%). Of the patients treated for oligoprogression, 5 patients (36%) had symptoms at the site of oligoprogressive disease, and radiation was thus palliative in nature as well. In the majority of cases, high-dose radiation was delivered to the primary site of disease (n = 23, 51%), and the remainder was delivered to metastatic sites including bone (n = 9, 20%), lymph nodes (n = 6, 13%), and other (n = 7, 16%). Almost half of patients (n = 22, 49%) received a treatment course of 5 fractions or less. The median time from NEN diagnosis to the start of high-dose radiation was 12 months (IQR 3–49); the median time from diagnosis to radiation was 18 months (IQR 8–91) for patients with well-differentiated disease, compared to 3 months (IQR 2–6) for patients with poorly-differentiated disease (p<0.001).

### Oncologic outcomes

At a median follow-up of 24 months (IQR 14–43), the 2-year actuarial rates of local relapse-free survival, new metastasis-free survival, progression-free survival (defined as the absence of local recurrence, development of new metastases, and worsening of any known metastases at the time of radiation), and overall survival after radiation were 98%, 45%, 41%, and 69%, respectively. Median progression-free survival after radiation was 19 months, and median overall survival after radiation was 43 months. Patients were additionally grouped by differentiation and stage, with oncologic outcomes and median time to progression in months summarized in Table 2.

**Table 2:** Median time to progression by differentiation and stage after high-dose radiation (n = 45).

	No evidence of active disease N (%)	Local progression at radiation site N (%), mTTP	New metastases N (%), mTTP	Progression of existing metastases N (%), mTTP
WD-L (n = 9)	6 (67%)	1 (11%), 28 mo	3 (33%), 26 mo	–
WD-M (n = 18)	7 (39%)	0 (0%), –	8 (44%), 9 mo	5 (28%), 5 mo
PD-L (n = 13)	4 (31%)	1 (8%), 5 mo	10 (77%), 8 mo	–
PD-M (n = 5)	1 (20%)	0 (0%), –	4 (80%), 3 mo	0 (0%), –
Total (n = 45)	18 (40%)	2 (4%), 16 mo	25 (56%), 9 mo	5 (22%)^a^, 6 mo

WD, well-differentiated; PD, poorly-differentiated; L, localized / locally advanced; M, metastatic; mTTP, median time to progression.

^a^Out of those with metastatic disease to start.

**Fig 1:**
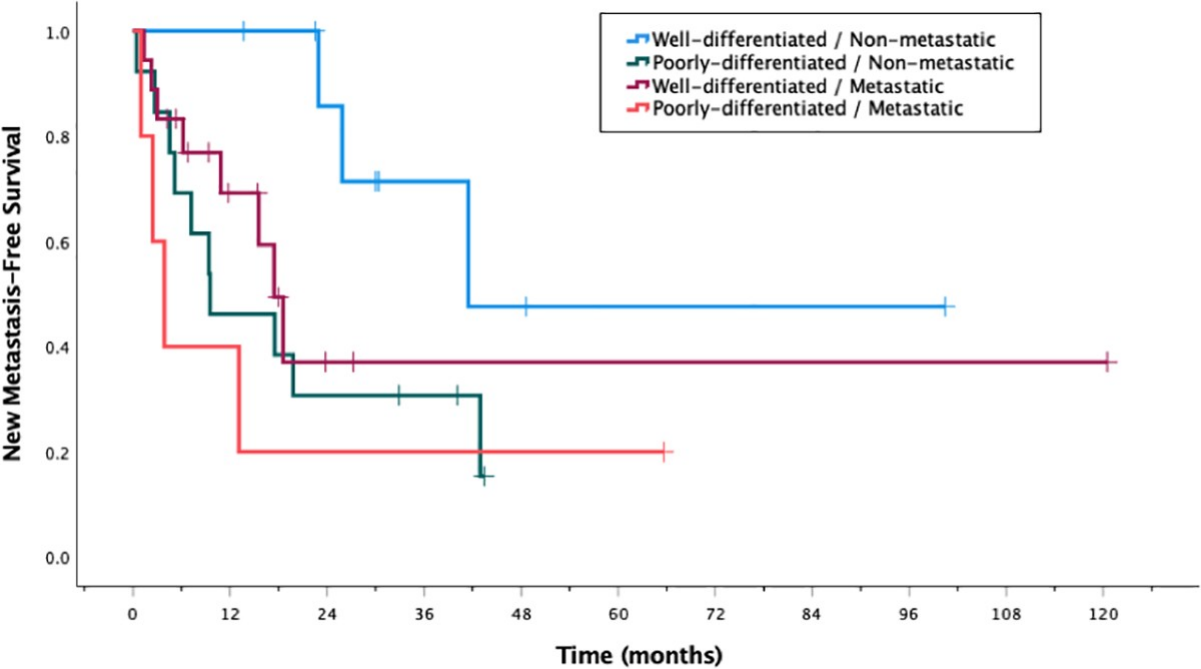
Kaplan-Meier plot of new metastasis-free survival after radiation, as grouped by differentiation and stage.

18 patients (40%) had no evidence of active disease at last follow-up, including 8 patients who had known metastatic disease at the time of radiation. Two patients (4%) had local progression at the site of radiation at a median time of 16 months (Table 3). Of these local recurrences, one was characterized by wall thickening and soft tissue nodularity in the region of the irradiated surgical bed that was not evaluable by RECIST, while the other initially achieved a partial response at 5 months after radiation per RECIST and later was found to have interval growth in the lesion 28 months after radiation. Both patients additionally developed new metastases at a median time of 23 months. In total, 25 patients (56%) developed new metastases after completion of radiation, including 33% (n = 3) of patients with WD-L disease, 44% (n = 8) of WD-M, 77% (n = 10) of PD-L, and 80% (n = 4) of PD-M, with progressively shorter median times to progression (26, 9, 8, and 3 months, respectively; p = 0.093) (Fig 1). Of the 23 patients with metastatic disease at the time of radiation, 5 (22%) had progression at their known untreated sites of distant disease at a median time of 6 months.

**Table 3:** Patient and disease characteristics of local recurrences in this study (n = 2).

	Primary site	Grade	Differentiation	Stage at RT	Intent of RT	RT site	Dose and fractionation	BED	Evaluable by RECIST	Time to local failure
1	Stomach	3	Poorly-differentiated	Locally advanced	Post-operative	Primary site (surgical bed)	45 Gy in 25 fx	53	No	4.5 months
2	Pancreas	1	Well-differentiated	Locally advanced	Definitive	Primary site	30 Gy in 5 fx	48	Yes	28 months

Acute and late toxicities after radiation were largely mild, self-resolving, and dependent on treatment location. Grade 3 toxicity was reported in only 2 patients (4%), both of whom developed acute mucositis/esophagitis requiring temporary feeding tube placement during radiation to primary head and neck cancers. There were no grade 4 or 5 acute or late toxicities.

The best response in the irradiated lesions was determined by RECIST for the 25 patients with treated lesions evaluable by RECIST. 28% (n = 7) had a complete response, 40% (n = 10) had a partial response, and 32% (n = 8) had stable disease at the irradiated site. No patients had disease that progressed through radiation. The best response was achieved at a median time of 4.2 months after radiation. On univariate analysis, age, primary site, histologic grade, tumor differentiation, baseline lesion size, stage, and BED were not associated with either a complete or partial response in the irradiated lesion.

On univariate analysis, age, primary site, stage, and BED were not predictive for local relapse or development of new metastasis. Factors associated with development of new metastasis were higher histologic grade (p = 0.007) and poorly-differentiated disease (p = 0.018), neither of which were predictive for local relapse.

## Discussion

While well-differentiated NENs have historically been thought of as radioresistant, there is now increasing evidence that radiation is effective in providing local control for both poorly-differentiated and well-differentiated disease. The present study contributes additional data to support the use of radiation for NENs, demonstrating an excellent rate of local control across various primary disease sites, grades, and tumor differentiation. While objective tumor response rates are limited to 10–15% with somatostatin analogues, 68% of patients treated with high-dose radiation in this study achieved either a partial or complete radiographic response in the treated lesion, suggesting a good level of radiosensitivity in these tumors [7–9].

For patients with localized NENs, the role of radiation may be to improve disease control after resection or provide a curative approach to unresectable tumors. Yet even for patients with metastatic disease, local control may provide an important role in stabilizing limited sites of distant disease. There were 8 patients in this study who were metastatic at the time of radiation and subsequently went on to achieve active disease-free status at a median follow-up of 14 months. All were treated to various sites of distant disease to a median BED of 72, and importantly, all but 1 had grade I/II well-differentiated tumors. Although it is possible that additional patients within this group would progress with longer follow-up, this subset highlights that radiation has the potential to at least delay a change in systemic therapy for well-selected patients.

Overall, local failure was an uncommon mode of failure after high-dose radiation in this study, occurring in only 2 out of 45 patients. Prior studies have reported a wide range of local failure rates after radiation from 10% to 30% at 2 years for various histologic subtypes, likely owing to the heterogeneity among both population and treatment delivered [11–13, 16, 19, 20]. In the 2 local failures in the present study, both patients received lower doses with BEDs of 43 and 58 to radiation targets in the abdomen, where higher doses may have been limited by the proximity of these sites to nearby critical structures. A prior study of external radiation for pancreatic NENs by Contessa et al identified a possible BED threshold of 49.6 Gy as being significant for local control, although notably all failures occurred in patients treated for liver metastases which received more palliative doses of radiation [21]. Thus, while radiation dose may be an important factor for local control of NENs, additional data is needed to conclusively support this association.

Despite a very low rate of local failure, the prevailing pattern of failure remains development of new metastases, the rate of which is dependent on well-established risk factors such as higher histologic grade and poorly-differentiated disease [3, 5]. Although the difference in new metastasis-free survival was not statistically significant among differentiation-stage groupings, distant failures appear to occur sooner and more frequently in patients with poorly-differentiated and metastatic disease at the time of radiation. Our 2-year metastasis-free survival rate of 45% is in concurrence with previously published studies [13, 19, 20].

As seen in this cohort, the predilection of NENs to progress systemically highlights the need for effective systemic therapy. For patients with gastroenteropancreatic NENs, such as the plurality of patients in the current study, PRRT represents an important therapeutic option at the time of progression. The landmark NETTER-1 trial reported a significant progression-free survival benefit and suggested an overall survival benefit for patients with well-differentiated advanced midgut NENs treated with ^177^Lu-Dotatate PRRT when compared to somatostatin analogues alone [10]. However, the potential for renal or hematologic toxicity may exclude certain patients from PRRT administration, and not all NENs demonstrate adequate somatostatin receptor expression for PRRT [22, 23]. Additionally, the degree of disease progression on interval imaging may vary widely from patient to patient, and treatment should be tailored to each individual’s burden of active disease. Thus, in patients who are not PRRT candidates or who present with low-volume progressive disease, high-dose radiation to limited targets remains an important consideration, and in some cases, this local control may help to preserve PRRT as a later-line therapy for more extensive disease progression.

There are several limitations to this study. The small sample size and heterogeneity of both disease and treatment characteristics are challenges inherent to studies on this population of patients. Follow up was often limited, as many patients were referred from outside hospitals and continued care at their referring institution shortly after radiation without many subsequent imaging records available in our system. There was no predefined schedule for intervals of follow-up imaging. Data on the use of systemic therapy either at the time of radiation or after was not readily available, and it is difficult to separate the effect of these therapies from that of the radiation course on oncologic outcomes, especially in terms of distant recurrences.

Radiation is an important treatment option to consider for patients with limited sites of unresectable disease from NENs and appears to be well-tolerated and highly effective in local control across the spectrum of histologies and disease presentations. The predominant mode of failure in this population remains distant metastasis, which is closely associated with histologic grade and tumor differentiation. Larger prospective trials with well-defined more homogeneous populations are needed to better establish the role of radiation in management of NENs.
